# Response: Commentary: Decomposition of Heart Rate Variability Spectrum into a Power-Law Function and a Residual Spectrum

**DOI:** 10.3389/fcvm.2018.00191

**Published:** 2019-01-17

**Authors:** Jane Kuo, Cheng-Deng Kuo

**Affiliations:** ^1^Department of Dentistry, Cathay General Hospital, Taipei, Taiwan; ^2^Division of Chest Medicine, Department of Internal Medicine, Changhua Christian Hospital, Changhua, Taiwan; ^3^Department of Medical Research, Taipei Veterans General Hospital, Taipei, Taiwan

**Keywords:** decomposition, heart rate variability, power-law function, residual spectrum, power spectral analysis, fractal

Dr. Paolo Castiglioni (1) comments on our paper “Decomposition of heart rate variability spectrum into a power-law function and a residual spectrum” (2). In that commentary, he pointed out that both the rg (= *s* · log(*Frq*) + *Y*) in Equation (1) in his commentary and the PSDrg have the units of PSD, or ms^2^/Hz, so that the spectral ratio rPSD in Equation (3) in his commentary is a dimensionless number. In the above argument, the “rg” denotes “regression,” “PSD” denotes “power spectral density,” and “rPSD” denotes “residual PSD.” We are grateful to Dr. Castiglioni for his careful examination of our equations. Unfortunately, the argument of Dr. Catiglioni was based on the misunderstanding caused by the omission of the units of the variables in our Equation (2). To avoid confusion and misunderstanding, we shall re-express our equations in the followings with the units of the variables placed in suitable positions in the equations.

To facilitate the decomposition of the PSD in the whole heart rate variability (HRV) spectrum, the power-law relation of HRV can be obtained by plotting $\log\left(\frac{PSD}{{\text{ms}}^{\text{2}}/\text{Hz}}\right)$ against $\log\left(\frac{Frq}{Hz}\right)$ within the frequency range from >0 Hz to the Nyquist frequency. Both $\frac{PSD}{\text{m}{\text{s}}^{2}/\text{Hz}}$ and $\frac{Frq}{Hz}$ are dimensionless. The 0 Hz point must be excluded because log(0) is not defined mathematically. The linear regression relation between $\log\left(\frac{\text{PSD}}{{\text{ms}}^{\text{2}}/\text{Hz}}\right)$ and $\log\left(\frac{Frq}{Hz}\right)$ in the PSD of HRV can be expressed as

(1) $$\log\left(\frac{PSD_{rg}}{{\text{ms}}^{\text{2}}/\text{Hz}}\right)=s\;\cdot \;\log\left(\frac{Frq}{Hz}\right)+Y$$

where the “log” denotes logarithm, the subscript “rg” denotes “regression,” and the “s” and “Y” are the “slope” and “Y-intercept” of linear regression between $\log\left(\frac{{\text{PSD}}_{\text{rg}}}{{\text{ms}}^{\text{2}}/\text{Hz}}\right)$ and $\log\left(\frac{Frq}{Hz}\right)$, respectively.

Equation (1) can be re-written as

(2) $$\frac{{\text{PSD}}_{\text{rg}}}{{\text{ms}}^{\text{2}}/\text{Hz}}=10^{s\cdot Log\left(\frac{\text{Frq}}{\text{Hz}}\right)+Y}=10^{Y}\cdot \;{\left(\frac{\text{Frq}}{\text{Hz}}\right)}^{\text{s}}.$$

Equation (2) shows that the $\frac{\text{PS}{\text{D}}_{\text{rg}}}{\text{m}{\text{s}}^{2}/\text{Hz}}$ is a power-law function of $\left(\frac{\text{Frq}}{\text{Hz}}\right)$. The difference between $\log\left(\frac{\text{PSD}}{{\text{ms}}^{\text{2}}/\text{Hz}}\right)$ and $\log\left(\frac{{\text{PSD}}_{\text{rg}}}{{\text{ms}}^{\text{2}}/\text{Hz}}\right)$ is the logarithm of the residual power spectral density, $\frac{\text{rPSD}}{\text{m}{\text{s}}^{2}/\text{Hz}}$, that cannot be accounted for by the $\frac{\text{PS}{\text{D}}_{\text{rg}}}{\text{m}{\text{s}}^{2}/\text{Hz}}$:

(3) $$\log\left(\frac{\text{rPSD}}{{\text{ms}}^{\text{2}}/\text{Hz}}\right)=\log\left(\frac{\text{PSD}}{{\text{ms}}^{\text{2}}/\text{Hz}}\right)-\log\left(\frac{{\text{PSD}}_{\text{rg}}}{{\text{ms}}^{\text{2}}/\text{Hz}}\right)=\log\left(\frac{\text{PSD}}{{\text{PSD}}_{\text{rg}}}\right).$$

Equations (2, 3) give immediately the following expression of rPSD:

(4) $$\text{rPSD}=\frac{\text{PSD}}{{\text{PSD}}_{\text{rg}}}\;\cdot \;({\text{ms}}^{\text{2}}\text{/Hz})=\text{PSD }\cdot \;10^{-\text{Y}}\cdot \;{\left(\frac{\text{Frq}}{\text{Hz}}\right)}^{-\text{s}}.$$

Since the PSDrg has the unit of ms^2^/Hz, the rPSD also has the same unit of ms^2^/Hz as that of PSD, according to Equation (4). Thus, the rPSD is not dimensionless, as asserted by Dr. Castiglioni (1). Because the rPSD is obtained from the original PSD by removing the power law constituent in it, its frequency components are all significantly smaller than those of the original PSD. This is comprehensible.

Castiglioni also commented on our fitting of the regression line up to the Nyquist frequency (1). He asserted that the PSDrg cannot be considered the true “fractal component” of the spectrum, because the regression slope *s* is influenced by the oscillations in the low-frequency (LF) and high-frequency (HF) bands. Though the PSDrg can be influenced by the oscillations in the LF and HF bands, its overall behavior is self-similar with respect to frequency because the PSDrg is a power-law function of frequency, according to Equation (2). It is doubtful that the PSDrg cannot be interpreted as a fractal. If the PSDrg cannot be interpreted as a “fractal,” then call it a power-law function of frequency. It is not important whether the power-law function PSDrg obtained in our decomposition method can be regarded as a fractal or not.

The purpose of decomposing the PSD of HRV into a power-law function of frequency and a residual PSD is to examine more closely the heart rate oscillations in the LF and HF regions, rather than to examine the frequency region below 0.04 Hz, because LF and HF regions are mostly concerned in many clinical settings that are associated with autonomic dysfunction of the patients. If we adhere to the international guidelines on HRV to analyze the PSD within the frequency range of <0.04 Hz (3–7), then the heart rate oscillations in the LF and HF regions cannot be examined in more details by using the decomposition techniques introduced by us in our previous article (2).

The PSD of HRV obtained from either short-term or long-term (6, 7) recordings of heart periods can be decomposed into a power-law function and a residual spectrum by using the mathematical technique devised by our group. The point is that if we are going to examine the LF and HF components of HRV in more details by using the decomposition method, then fitting the regression line between $\log\left(\frac{\text{PSD}}{{\text{ms}}^{\text{2}}/\text{Hz}}\right)$ and $\log\left(\frac{\text{Frq}}{\text{Hz}}\right)$ beyond the upper frequency limit (0.04 Hz) of the very low frequency (VLF) component is a necessity. Otherwise, the PSD within the frequency range of LF and HF will not be decomposed, and the LF and HF components of HRV cannot be examined in more details by using the decomposition method developed by our group. Thus, the decomposition of the PSD of HRV within the whole frequency range from >0 to the Nyquist frequency is the simplest way of performing the decomposition analysis.

## Author Contributions

JK: drafting of the manuscript. C-DK: conception, design and finalization of the work.

### Conflict of Interest Statement

The authors declare that the research was conducted in the absence of any commercial or financial relationships that could be construed as a potential conflict of interest.

## References

[1] Castiglioni P. Commentary: decomposition of heart rate variability spectrum into a power-law function and a residual spectrum. Front Cardiovasc Med. (2018) 5:94. 10.3389/fcvm.2018.00094 30094240 PMC6070638

[2] Kuo J Kuo CD. Decomposition of heart rate variability spectrum into a power-law function and a residual spectrum. Front Cardiovasc Med. (2016) 3:16. 10.3389/fcvm.2016.00016 27314001 PMC4889601

[3] Kobayashi M Musha T. 1/f fluctuation of heartbeat period. IEEE Trans Biomed Eng. (1982) 29:456–7. 7106796 10.1109/TBME.1982.324972

[4] Otsuka K Nakajima S Yamanaka T Vagal tone and its association with a new index of heart rate variability called 1/f fluctuations. J Ambul Monit. (1994) 7:213–18.

[5] Task Force of the European Society of Cardiology and the North American Society of Pacing and Electrophysiology Heart rate variability. Standards of measurement, physiological interpretation, and clinical use. Eur Heart J. (1996) 17:354–81. 8737210

[6] Sassi R Cerutti S Lombardi F Malik M Huikuri HV Peng CK. Advances in heart rate variability signal analysis: joint position statement by the e-Cardiology ESC Working Group and the European Heart Rhythm Association co-endorsed by the Asia Pacific Heart Rhythm Society. Europace (2015) 17:1341–53. 10.1093/europace/euv015 26177817

[7] Kazuma N Nozaki M Nakamura E Matsuoka I Otani T Biological rhythm in 1/f fluctuations of heart rate in asthmatic children. Allergol Intern (2004) 53:265–9. 10.1111/j.1440-1592.2004.00343.x

